# Leydig Cell Tumor-Induced Gonadotropin-Independent Precocious Puberty Progressing to Gonadotropin-Dependent Precocious Puberty Post Orchiectomy: Out of the Frying Pan Into the Fire

**DOI:** 10.7759/cureus.21165

**Published:** 2022-01-12

**Authors:** Pankaj Singhania, Rana Bhattacharjee, Partha Pratim Chakraborty, Subhankar Chowdhury

**Affiliations:** 1 Endocrinology and Metabolism, Institute of Post Graduate Medical Education and Research, Kolkata, IND; 2 Department of Endocrinology, Medical College Kolkata, Kolkata, IND

**Keywords:** gnrh, testosterone, ultrasonography, testis, leydig cell tumor, puberty, precocious

## Abstract

Many pathologies can cause gonadotropin-independent precocious puberty (GIPP) in prepubertal boys. Leydig cell tumor is one rare cause of this presentation. Here we present a six-year-old boy with features of isosexual precocious puberty, high testosterone levels, low gonadotropin levels, and bone age advancement. Testicular USG revealed a left-sided testicular tumor. The left testis was removed surgically, and the Leydig cell tumor was confirmed on histopathology. Post orchiectomy, the boy had elevated testosterone levels with raised luteinizing hormone (LH) levels. A diagnosis of gonadotropin-dependent precocious puberty (GDPP) was made. He has been initiated on monthly gonadotropin-releasing hormone (GnRH) agonist therapy.

## Introduction

Testicular tumors account for 2-4% of cancers in children [1]. A bimodal age distribution for the incidence of testicular tumors has been observed; one peak occurs in the first two years of life, and the second occurs in young adulthood. Cryptorchidism and gonadal dysgenesis are the two most important risk factors associated with testicular tumors in children [2-3]. Prepubertal teratomas (50%) and yolk sac tumors account for most pediatric testicular tumors. Epidermoid cysts (15%) and stromal tumors (Leydig and Sertoli cell tumors) comprise the rest (10%) [4-5]. Leydig cell tumors, which account for 3-6% of testicular tumors in prepubertal boys, are the most common hormone-secreting testicular tumors [6]. As a result, the most common presentation of these tumors in boys between 5 and 10 years of age is peripheral precocious puberty [7]. These boys present with gonadotropin-independent precocious puberty (GIPP) with high androgen levels and low levels of gonadotropins. After successful surgical removal of the tumor, some may progress to gonadotropin-dependent precocious puberty (GDPP) with the reappearance of signs of puberty because of reactivation of a primed hypothalamic-pituitary-gonadal axis.

## Case presentation

A six-year-old boy, younger of two siblings, born out of a non-consanguineous marriage, presented to our outpatient department with chief complaints of appearance of secondary sexual characteristics, phallic enlargement, and height acceleration over the last 4-6 months. In addition, the boy started developing pubic hair growth, facial hair growth, and enlargement of the penis, which progressed rapidly over the duration of the illness. There was no history of seizures, headache, episodes of unconsciousness, or any other features suggestive of raised increased intracranial pressure (ICP). There was no history of radiation exposure or childhood malignancy. There was no history of hyperpigmentation or other features suggestive of adrenal insufficiency. There was no exposure to testosterone in the past. He was born full-term by normal vaginal delivery, and his perinatal history was unremarkable. There was no history of precocity in the family and the elder sister (10 years old) had no medical issues.

**Figure 1:**
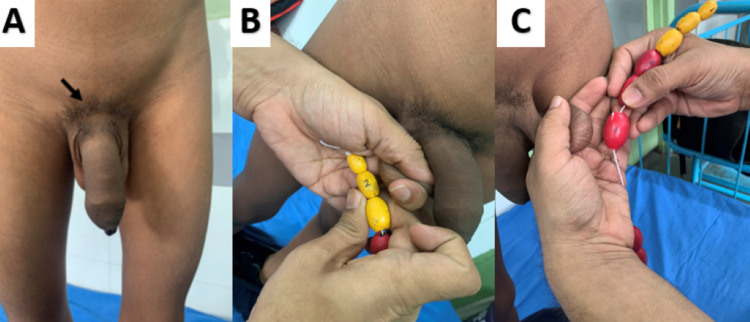
Clinical picture showing penile enlargement and Tanner stage 3 pubic hair (A). Note the asymmetric testicular volume; right (2 ml) (B) being smaller than the left (6 ml) (C).

On examination, the boy was healthy-looking with a height of 129 cm (+2.5 SDS, Indian Academy of Pediatrics [IAP] 2015 growth charts) and a weight of 22 Kg. His pubic hair (Figure 1A) was Tanner stage 3. The right testicular volume was 2 ml (Figure 1B), and the left testis was 6 ml (Figure 1C) by Prader orchidometer, and they were firm in consistency. The stretched penile length was 12 cm which was greater than the 95th percentile for age (median at this age is 6.0 cm, 5th-95th percentile: 4.2-7.2 cm). There was no genital hyperpigmentation.

The following initial investigations were obtained (Table 1).

**Table 1:** Baseline and follow-up hormonal parameters. LH: Luteinizing hormone; FSH: Follicle-stimulating hormone; TSH: Thyroid-stimulating hormone; T4: Thyroxine.

Parameter	Value at diagnosis	4 weeks post-surgery	Reference range
Serum LH	0.1 U/L	3.7 U/L	0.3-0.6
Serum FSH	0.2 U/L	3.5 U/L	<1-3
Serum fasting testosterone	293 ng/dl	28.4 ng/dl	<20
Serum TSH	2.05 mcIU/ml		0.340-6.00
Serum-free T4	1.43 ng/dl		0.8-1.9

**Figure 2:**
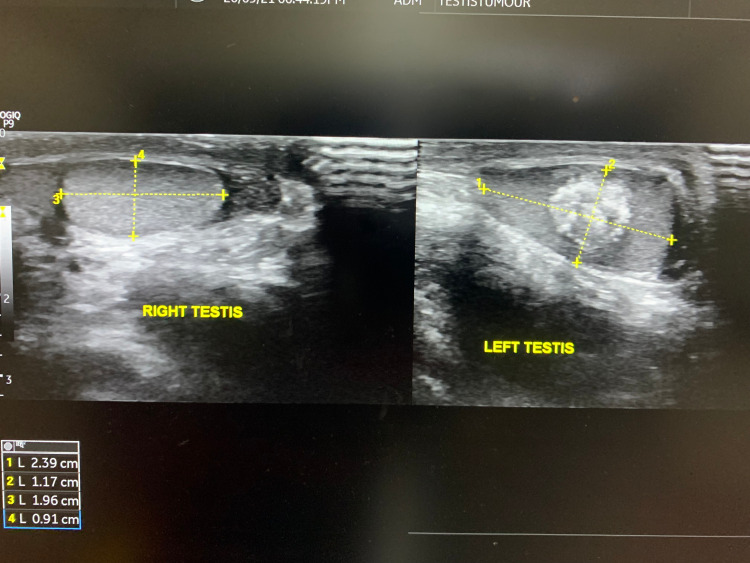
Enlarged left testes with centrally located, hyperechoic lesion with lobulated margin and normal right testes on USG.

**Figure 3:**
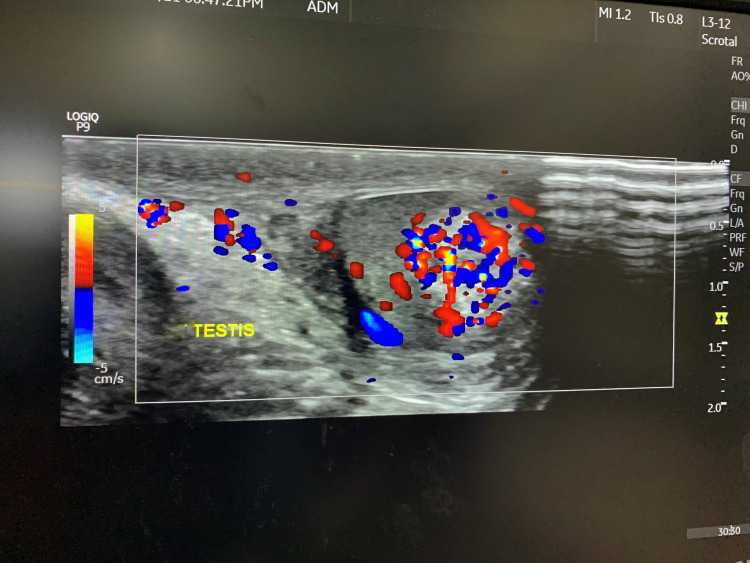
Hypervascular left testes mass on Doppler study.

**Figure 4:**
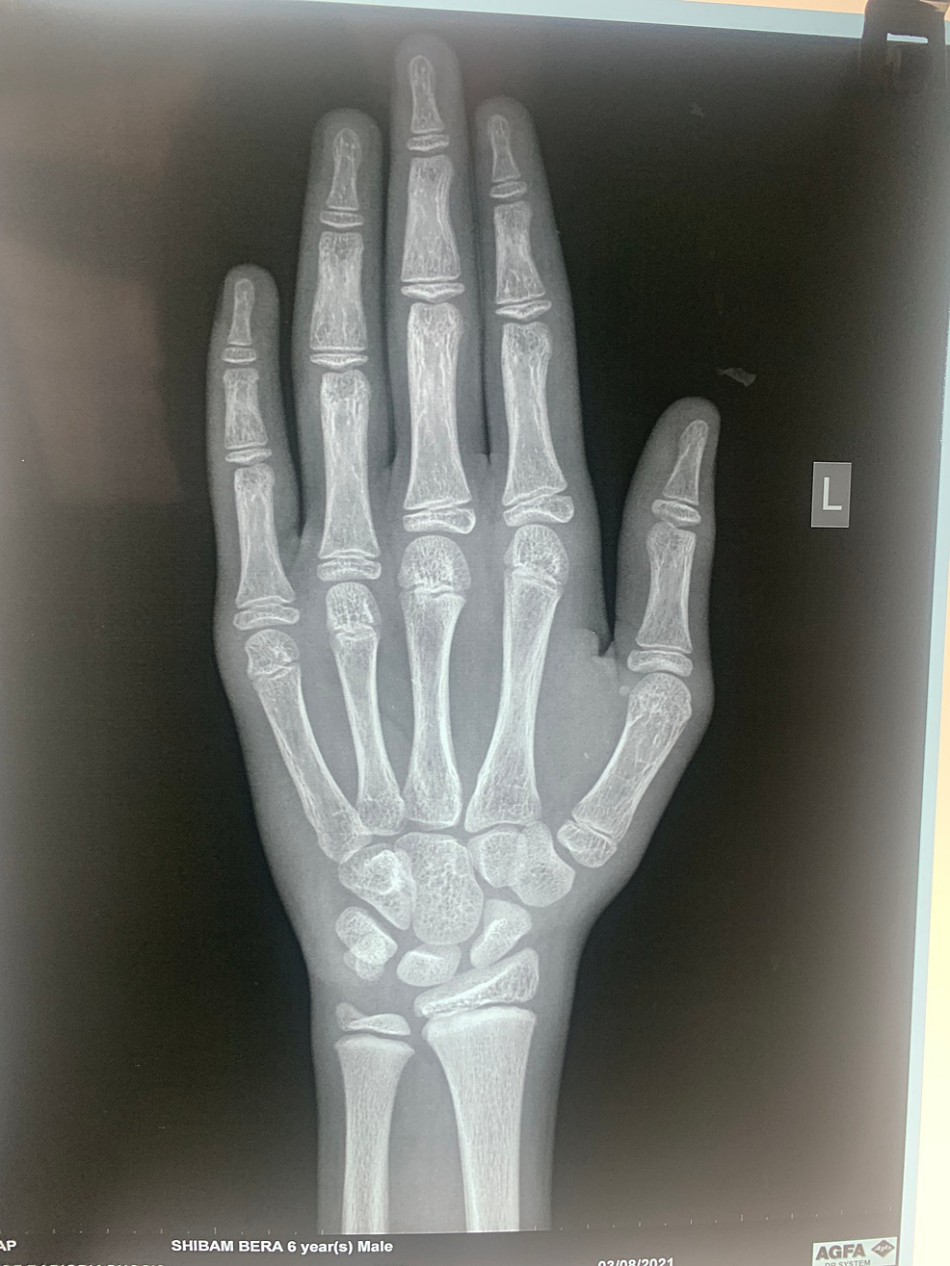
X-ray of left hand showing bone age of 12 years by Greulich and Pyle chart.

Considering the asymmetrical testicular size, a USG of testes was undertaken, which showed that the left testis was enlarged (2.4*1.2 cm) compared to the right (1.9*0.9 cm) (Figure 2). The left testis showed a 1*1 cm heterogeneously hyperechoic, centrally located, space-occupying lesion, with increased vascularity on Doppler study (Figure 3). Both the epididymis were normal. X-ray of the left hand showed a bone age of 12 years (Figure 4).

With a diagnosis of peripheral precocious puberty due to a hormone-secreting testicular tumor, the following investigations were further obtained (Table 2)​​​​​​.

**Table 2:** Tumor markers. B-HCG: Beta subunit of human chorionic gonadotropin; AFP: Alpha feto protein; LDH: Lactate dehydrogenase.

Parameter	Value	Reference range
Serum B-HCG	<0.6 mIU/ml	<2
Serum AFP	1.52 ng/ml	<12
Serum LDH	268 U/L	120-300

Though a diagnosis of congenital adrenal hyperplasia (CAH)-induced testicular adrenal rest tumor (TART) was less likely, to exclude CAH, baseline investigations were obtained, and a cosyntropin stimulation test was planned (Table 3).

**Table 3:** Basal and cosyntropin stimulated values. ACTH: Adrenocorticotrophic hormone; DHEAS: Dehydroepiandrosterone sulfate; 17OHP: 17-hydroxyprogesterone.

Parameter	Baseline	60 minutes after cosyntropin	Reference
Plasma ACTH	29.4 pg/ml		<46
Serum Cortisol	4.43 mcg/dl	22.6 mcg/dl	5-25
Serum DHEAS	20.9 mcg/dl		0-44
Serum 17OHP	1.49 ng/ml	2.55 g/ml	0.03-0.9

**Figure 5:**
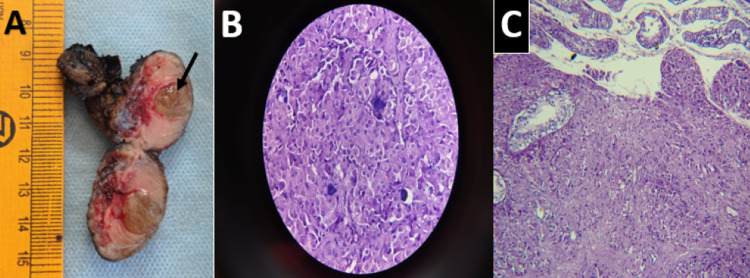
Gross cut section of the left testis (4 X 3.5 X 3 cm) containing well-circumscribed yellow-colored nodule (arrow) measuring 2 cm (A). The nodule is composed of polygonal cells with abundant granular cytoplasm, round nuclei resembling Leydig cells. Large areas of calcification, including psammomatous calcification (dark blue areas in B), are also seen. These features are suggestive of Leydig cell tumor (B: X400; C: X100).

MRI of the hypothalamic-pituitary area done prior to visiting us was normal. Based on the above findings, a final diagnosis of the testicular tumor, probably Leydig cell tumor causing GIPP, was made. The urology team performed a high inguinal orchiectomy under general anesthesia. Histopathology confirmed a diagnosis of testicular Leydig cell tumor (Figure 5A-5C).

He continued to recover well post-surgery. At four weeks follow-up, the boy had the appearance of new pubic hair on examination. His right testes increased to 4 ml (preoperatively 2 ml). He had persistently elevated testosterone level, and the luteinizing hormone (LH) level was found to be raised (Table 1). Our worst fear had come true; the boy now cured of GIPP had landed in GDPP. He was initiated on injection leuprolide 3.75 mg monthly and is being followed up for pubertal progression, growth, and development. Four months after surgery and having received three monthly leuprolide injections, his current height is 131 cm.

## Discussion

Isosexual precocious puberty in boys is diagnosed by penile enlargement, the appearance of pubic hair, deepening of voice, and height acceleration before nine years of age. Precocious puberty can be of two types; central and peripheral (GIPP). Central precocious puberty is caused by premature activation of the hypothalamic-pituitary-gonadal axis. On the other hand, GIPP is caused by various causes like congenital adrenal hyperplasia, familial male-limited testotoxicosis, exposure to exogenous androgens, HCG-secreting tumor, and adrenal and testicular tumors [8-9].

Our patient presented with GIPP due to a testicular Leydig cell tumor. Leydig cell tumors in childhood are most commonly recognized when they present with precocious puberty with testicular enlargement or a testicular mass, high testosterone levels, and low gonadotropin levels. USG is the investigation of choice in such cases. It generally presents as an isolated solid, hypoechoic mass located in the periphery of the testis [10-11]. Color Doppler shows a hyper-vascular lesion [10]. Tumor markers can help exclude other causes. 

Several cases of Leydig cell tumors in boys have been reported in the past [12]. Most of them had a similar presentation of penile enlargement and pubic hair growth. Scrotal USG was the primary diagnostic modality in cases where a mass was not palpable [13]. Treatment options include radical orchiectomy or a more conservative testis sparing surgery. USG and tumor markers can help us decide the modality of surgery [14]. However, long-term follow-up, including conversion to GDPP, has not been reported in most cases. It is expected that the patient will be cured of pubertal advancement post-surgery as the source of excess age-inappropriate androgen has been removed. One month after surgery, the testosterone level of our patient was above the normal prepubertal range, and the basal LH level reached the pubertal level. This is likely due to the priming effect of high circulating testosterone on the hypothalamic-pituitary-gonadal axis, which subsequently got prematurely activated because of a sudden drop of the testosterone level following the surgery [15]. There are few reported cases of central precocious puberty after the treatment of testicular Leydig cell tumors, mostly 3-6 months after orchiectomy [16-17]. In our case, the rise in gonadotropins could be detected as early as four weeks after surgery due to vigilant early follow-up. A monthly injection of leuprolide 3.75 mg was started in our patient. The follow-up plan was to monitor the pubertal feature and the periodic serum testosterone and LH estimation.

## Conclusions

Leydig cell tumor is a rare cause of GIPP in prepubertal boys. It should be suspected in boys presenting with GIPP with or without a palpable testicular mass. Surgical excision is the treatment of choice. Post-surgery surveillance should include looking for progression to GDPP. This might occur because sex steroid directly affects the hypothalamus and can accelerate the onset of centrally mediated puberty. This becomes apparent once the testosterone levels are lowered post-surgery. This report described a case of GIPP due to a Leydig cell tumor, which converted to GDPP following removal of the tumor.
